# To Dr. Haighton

**Published:** 1801-10

**Authors:** J. Primrose

**Affiliations:** Wrentham


### To Dr. HAIGHTON.

Dear Sir,

YOUR'S of the 3d inftant informed me of your not being able to find my firft letter, for which reafon you have requefted a recapitulation, with an addition of fome obfervations in my laft. The woman who affords the following fubject, before fhe became pregnant, laboured under a partial prolapfus uteri, the appearances of which gradually fubfided when fhe became pregnant; and during her latter months complained of nothing more than forcing pains towards the os-facrum; (I ought to obferve that this woman was of a very weak conftitution, and that this child was her tenth) her labour came on fuddenly, was a breech prefentation, but the natural pains were quite fufficient for its completion. After waiting the ufual time after delivery, and no pains following, I was induced to examine; upon which, to my furprife, I found a confiderable protrufion of the uterus, which led me to fend for my father, who advifed me to attempt the extraction of the placenta by introducing my hand, with a view to extricate it; at the fame time my hand affifted the return of the uterus, by preffing at the fundus, during the time that my left hand was affifting the feperation of the placenta at the funis. From there being fuch a confiderable adhefion of the placenta, I did not fucceed in obtaining all of it the firft time; on proceeding to make the fecond attempt (which we thought juftifiable, not only for the obtaining what remained of the placenta, but with a further view that the return of the uterus might be effected by the hand being carried to the fundus) to my great aftonifhment I found a defcent of the whole body of the uterus; after this the patient gradually funk: however, attempts were immediately made for its reduction, as far as were thought prudent, as the woman appeared almoft exhaufted. She did not revive fufficient to encourage me to make any further attempts till gangrene had made its appearance, which was in the fpace of forty-eight hours.

Had this woman not funk fo immediately after the fecond defcent, I am fenfible I could not have fucceeded in the reduction; not only from the protrufion being fo confiderable, but

 from from the firmnefs, or tenfion, being fo great as not to yield to compreffion. Mortification and putrid fever increafed rapidly; fomentations, &c. were ordered, and the patient was fupported with bark, and every requifite cordial, and fomentations were continued till fuppuration took place, which was complete, in the greateft portion of the prolapfed part, in feven or eight days; what remained, difcharged a great deal, and gradually wafted, as her health improved, and what remained at laft fpontaneoufly retracted.

It is five or fix months fince this woman was under my care; and I have now the fatisfaction of adding that fhe enjoys much better health than before fhe became pregnant.

I hope, dear Sir, if you fee any impropriety in the above ftatement, that you will make what alterations you conceive neceffary. I remain, your's, &c.

*Wrentham, Aug.* 14, 1801. J. PRIMROSE.

